# Reduced expression of intercellular adhesion molecule-1 in ovarian adenocarcinomas

**DOI:** 10.1054/bjoc.2001.2075

**Published:** 2001-09-01

**Authors:** J M Arnold, M Cummings, D Purdie, G Chenevix-Trench

**Affiliations:** The Queensland Institute of Medical Research, PO Box Royal Brisbane Hospital, Herston, Queensland, 4006, Australia; Department of Pathology, University of Queensland, St Lucia, Brisbane, 4067, Austrlia

**Keywords:** ovarian adenocarcinoma, ICAM-1, 5-aza-2′-deoxycytidine, loss of heterozygosity, cDNA array

## Abstract

Ovarian adenocarcinomas develop as the result of multiple genetic and epigenetic changes in the precursor ovarian surface epithelial (OSE) cells which result in a malignant phenotype. We investigated changes in gene expression in ovarian adenocarcinoma using a cDNA array containing 588 known human genes. We found that intercellular adhesion molecule-1 (ICAM-1) was expressed at lower levels in the ovarian tumour cell lines OAW42, PEO1 and JAM than in the immortalised human ovarian surface epithelial cell line HOSE 17.1. Further investigation revealed ICAM-1 was expressed in the surface epithelium of normal ovaries and both mRNA and protein expression levels were reduced in the majority of ovarian adenocarcinoma cell lines and primary tumours. ICAM-1 expression was increased in 8/8 cell lines treated with the de novo methyltransferase inhibitor 5-aza-2′-deoxycytidine, indicating that methylation of CpG islands may play a role in the down-regulation of its expression in primary tumours. There was a significant association between patients whose tumours expressed ICAM-1 and survival (*P*= 0.03), suggesting that expression levels of ICAM-1 may have clinical relevance. © 2001 Cancer Research Campaign


British Journal of Cancer (2001) 85(9), 1351–1358
© 2001 Cancer Research Campaign
doi: 10.1054/ bjoc.2001.2075, available online at http://www.idealibrary.com on IDEAL

http://www.bjcancer.com

### Reduced expression of intercellular adhesion molecule-1 in ovarian adenocarcinomas

**JM Arnold[1], M Cummings[2], D Purdie[1] and G Chenevix-Trench[1,2]**

[1]The Queensland Institute of Medical Research, PO Box Royal Brisbane Hospital, Herston, Queensland, Australia, 4006; [2]Department of Pathology, University of Queensland, St Lucia, Brisbane, Australia, 4067

**Summary** Ovarian adenocarcinomas develop as the result of multiple genetic and epigenetic changes in the precursor ovarian surface epithelial (OSE) cells which result in a malignant phenotype. We investigated changes in gene expression in ovarian adenocarcinoma using a cDNA array containing 588 known human genes. We found that intercellular adhesion molecule-1 (ICAM-1) was expressed at lower levels in the ovarian tumour cell lines OAW42, PEO1 and JAM than in the immortalised human ovarian surface epithelial cell line HOSE 17.1. Further investigation revealed ICAM-1 was expressed in the surface epithelium of normal ovaries and both mRNA and protein expression levels were reduced in the majority of ovarian adenocarcinoma cell lines and primary tumours. ICAM-1 expression was increased in 8/8 cell lines treated with the de novo methyltransferase inhibitor 5-aza-2′-deoxycytidine, indicating that methylation of CpG islands may play a role in the down-regulation of its expression in primary tumours. There was a significant association between patients whose tumours expressed ICAM-1 and survival ($P$ = 0.03), suggesting that expression levels of ICAM-1 may have clinical relevance. © 2001 Cancer Research Campaign

**Keywords**: ovarian adenocarcinoma; ICAM-1; 5-aza-2′-deoxycytidine; loss of heterozygosity; cDNA array

Adhesion molecules play an integral role in tumour growth, invasion and metastasis and have been shown to influence the host immune response to malignant cells. Tumour progression is associated with changes in the expression of cellular adhesion molecules which accompany the disaggregation of individual cells from the primary tumour and their penetration through the basement membrane, migration through the blood or lymph vessels, and attachment to a distant site (Johnson, 1991). Adhesion molecules are also required to provide ‘co-stimulatory’ antigen-independent interactions between leukocytes and target cells in the initiation of a cellular immune response against tumour cells (Springer, 1990).

Intercellular adhesion molecule-1 (ICAM-1 CD54) is a single-chain, 90 kDa inducible cell-surface glycoprotein and a member of the immunoglobulin supergene family which mediates many adhesion-dependent cell–cell interactions. It is constitutively expressed on haemopoietic cells such as tissue macrophages, monocytes, B-cells, activated T-cells and germinal centre dendritic cells. ICAM-1 expression can also be induced by inflammatory cytokines such as IFN-γ, IL1-β and TNF-α in non-haemopoietic cells including vascular endothelium, thymic epithelium and dermal fibroblasts (Springer et al, 1987; Springer, 1990). ICAM-1 and -2 and their counter-receptors, the cell-surface integrins lymphocyte-function-associated antigen-1 (LFA-1) and the complement receptor type-3 (CR-3, Mac-1, CD11a/CD18) have been shown to be essential for most cell–cell interactions in the immune response (Diamond et al, 1991), including MHC-restricted antigen presentation, cell proliferation induced by antigens and mitogens, T-cell mediated cytotoxicity and transvascular lymphocyte migration.

Received 5 February 2001
Revised 16 July 2001
Accepted 23 July 2001

Correspondence to: Dr J Arnold

Ovarian adenocarcinoma is the sixth most common cancer among women worldwide, and the leading cause of death from gynaecological neoplasias (Parkin et al, 1999). Ovarian adenocarcinomas arise from the surface epithelium of the ovary (OSE), but the molecular events that drive the initiation and progression of ovarian adenocarcinoma are not well defined. The lack of symptoms during the early stages of the disease results in the majority of women presenting with advanced tumours that do not respond well to treatment. We investigated changes in the expression of a panel of 588 known human genes in ovarian adenocarcinoma cell lines compared with immortalised human ovarian surface epithelial (HOSE) cell using a cDNA array (Arnold et al, 2001). This revealed that ICAM-1 expression was decreased in ovarian adenocarcinoma cell lines compared with the HOSE cells. The aim of the present study was to investigate the extent of the down-regulation of ICAM-1 expression in a larger panel of ovarian adenocarcinoma cell lines and primary tumours at both the RNA and protein levels, and to determine whether expression was related to patient survival.

### METHODS

### Cell lines, ovarian surface epithelium cultures and primary tumours

Human ovarian surface epithelial cell lines (HOSE) 17.1 and 1.1, immortalised with a retroviral vector expressing human papillomavirus oncogenes (Tsao et al, 1995) were maintained in a medium composed of 1:1 M199:MCDB105 with 10% FCS. The HEY ovarian adenocarcinoma cell line was maintained in MEMα medium with 10% FCS (Buick et al, 1985). The remaining ovarian adenocarcinoma cell lines–OAW 42 (Wilson, 1984), PEO1 (Wolf et al, 1987), PEO14 (Langdon et al, 1988), JAM (Ward et al,

1987), SKOV3 (Fogh and Trempe, 1975), COLO316 (Woods et al, 1979), CAOV3 (Wong et al, 1999), OVCAR-3 (Hamilton et al, 1983) and 27/87 (derived by T Hurst) – were all maintained in RPMI 1640 with 10% FCS. Cells were harvested for RNA isolation at about 80% confluence. The cell lines OAW 42, PEO1, PEO14, JAM, SKOV3 and COLO316 were derived from serous tumours, and 27/87 was from an endometrioid tumour. The histological types of the tumours from which the cell lines HEY, CAOV3 and OVCAR3 were derived are unknown.

Primary cultures of human ovarian surface epithelial (OSE) cells were prepared according to the method of Kruk et al (1990). Epithelial cells were obtained by scraping contaminating stromal cells away from proliferating epithelial sheets and were cultured in 1:1 MCDB105: Medium 199 with Earles' salts, supplemented with 20 ng $ml^{-1}$ epidermal growth factor, 400 ng $ml^{-1}$ hydrocortisone and 15% fetal calf serum. Their distinctive cellular morphology was used to confirm that the cultures were epithelial cells. In one case, RNA was extracted directly from the peeled epithelial cells without culturing.

Ovarian adenocarcinomas were obtained from 44 patients undergoing surgery. There were 35 serous, 6 endometrioid, one mucinous and 2 clear-cell tumours. There were 43 malignant tumours and the other was of low malignant potential (LMP). All patients were staged at laparotomy, in accordance with the recommendations of the International Federation of Gynaecology and Obstetrics (FIGO). Of the malignant tumours, one was FIGO stage 1, 4 stage 2, 33 stage 3 and 5 stage 4. There were two grade 1–2, 11 grade 2, 10 grade 2/3, 17 grade 3, one grade 3–4, and 2 that were not graded. Clinicopathological data for these tumours are summarised in Table 2. Constitutional DNA was available in all cases from peripheral blood. RNA was available from a subset of 34 tumours.

### Isolation of DNA and RNA

Tumour tissue was dissected free from necrotic tissue and connective tissue, and mechanically dispersed prior to collagenase treatment (0.1 mg $ml^{-1}$ in Hanks balanced salt solution). Erythrocytes and necrotic cells were removed with ficoll-paque, and genomic DNA was extracted by the salting-out method as described elsewhere (Chenevix-Trench et al, 1992). Total RNA was extracted from fresh primary tumours and sub-confluent cultured cell lines using Tri-reagent (Sigma), following the manufacturer's instructions. PolyA + RNA was prepared from total RNA using Dynabeads (Dynal) according to the recommended protocol.

### Northern blot analysis

RNA was denatured and electrophoresed on a formaldehyde-agarose gel and transferred to a nylon membrane (Amersham Hybond N+) by capillary blotting overnight according to standard protocols (Sambrook et al, 1989). The RNA was then fixed to the membrane by UV irradiation. Probes were prepared from RT-PCR products by random priming (Amersham Megaprime) and hybridization was carried out for 2 hours in ExpressHyb solution (Clontech. Inc.) at 65˚C (for random primed probes) before a standard washing procedure.

### Semi-quantitative RT-PCR

cDNA synthesis was performed on 1 µg of total RNA in a volume of 20 µl using SuperScript II reverse transcriptase (Gibco BRL) according to the manufacturer's protocol. PCR was carried out on 1 µl of this cDNA in a volume of 20 µl incorporating $^{33}$P-dATP using standard PCR cycling conditions. To ensure quantitation was assessed only on amplified cDNA, all primer pairs were designed to span at least one intron. The ICAM-1 primers used for the 5-aza-2′-deoxycytidine treated cell lines (CCAACCTCACCGTG-GTGCTGCTCCG and GCAGTGTCTCCTGGCTCTGGTTCC) amplify a 479 bp product from cDNA. The ICAM-1 primers used for the primary tumours and cell lines (AAGCTTGCAAC-CTCAGCCTCGCTATGG and CGGAGCAGCACCACGGT-GAGGTTGG) amplify a 480 bp product from cDNA. The β-actin primers used (CGTGACATTAAGGAGAAGCTGTGC and CTCAGGAGGAGCAATGATCTTGAT) amplify a 375 bp product from cDNA. Multiplex PCR was carried out and 5 µl of product removed at cycles 25, 29, 33 and 37 and the products run on a denaturing acrylamide gel prior to autoradiography. The autoradiogram showing products after 29 cycles of amplification was scanned and analysed by densitometry for band intensity. The value for the ICAM-1 band was then expressed as a percentage of the corresponding band for β-actin after subtracting background values. The ICAM-1 value for the HOSE 17.1 internal control was then adjusted to 100 and all other values adjusted accordingly.

### Immunohistochemistry

Normal ovaries (5) and ovarian adenocarcinomas (20) were embedded in OCT compound (Miles Inc, USA), snap frozen in liquid nitrogen, and stored at –70˚C until sectioning. Cultured cell lines were harvested by scraping, were washed in PBS and embedded as a dense suspension in OCT and frozen as above. Serial sections (7 µm) were air-dried and fixed in acetone at room temperature for 10 minutes. After rehydration with tris-buffered saline (TBS), pH 7.4, endogenous peroxidase activity was blocked by incubation in 1% $H_2O_2$ 0.1% $NaN_3$ in TBS for 10 minutes, followed by blocking non-specific antibody binding by incubation in 4% skim milk powder in TBS for 15 minutes and normal goat serum for 20 minutes. The anti-ICAM-1 monoclonal antibody (Immunotech clone 84H10) was then applied overnight at 0.2 µg $ml^{-1}$. The slides were then incubated in biotinylated goat anti-rabbit immunoglobulin (Zymed Corp, San Francisco) for 15 minutes, followed by streptavidin horseradish peroxidase (Zymed) for 8 minutes. Slides were washed 3 times in TBS for 5 minutes between all steps. One serial section was processed and stained as above but the primary antibody was omitted as a negative control. Finally, the sections were incubated with 3,3′-diaminobenzidine (0.05%) and $H_2O_2$ (0.18%) in tris-saline buffer for about 5 minutes to produce a brown reaction product and the sections were counterstained with haematoxylin, dehydrated in alcohol, cleared in xylene and mounted. Staining of vascular endothelium was monitored as a positive control.

### 5-aza-2′-deoxycytidine treatment of ovarian adenocarcinoma cell lines

Cell lines were plated at 20–30% confluence and treated 24 hours later (day 0) with 0, 0.5 or 2.0 µM 5-aza-2′-deoxycytidine. Fresh media containing the same concentration of 5-aza-2′-deoxycytidine was added on day 2 and cells were harvested for RNA extraction on day 5.

 *© 2001 Cancer Research Campaign*

### Loss of heterozygosity (LOH) analyses

LOH was assessed at 19p13 with the D19S558 marker. PCR amplification was carried out for 35 cycles in the presence of $^{33}$P-dATP and PCR products were analysed on a denaturing polyacrylamide gel. LOH was scored conservatively as a substantial reduction in the intensity of one allele by 2 independent observers one of whom was blind with respect to the sample identity.

### Statistical analyses

Survival curves, survival probabilities and estimated mean survival times were calculated according to the Kaplan–Meier method. The differences between the survival curves for the ICAM-1-positive and ICAM-1-negative groups were evaluated using the log-rank test. This was evaluated in 4 different ways. Expression was assigned positive or negative by immunohistochemistry according to whether there was greater than or less than 10% of tumour cells staining and whether there was greater than or less than 25% of tumour cells staining. Expression was also assigned positive or negative by RT-PCR according to whether there was greater than or less than 1% that of HOSE 17.1 band intensity and whether there was greater than or less than 10% that of HOSE 17.1 band intensity. Fisher's exact test was used to evaluate the association between ICAM-1 expression and LOH at 19p13 and the test for trend was used to examine for a relationship between ICAM-1 expression and stage or grade.

### RESULTS

### Analysis of ICAM-1 mRNA expression in ovarian cancer cell lines and primary tumours

We have previously used a cDNA array containing 588 known genes to compare gene expression in 3 ovarian adenocarcinoma cell lines with a HOSE cell line (Arnold et al, 2001), which showed that ICAM-1 expression was reduced in the ovarian adenocarcinoma cell lines compared with the HOSE cell line. To confirm this result, we performed semi-quantitative RT-PCR on a series of 10 ovarian adenocarcinoma cell lines, an immortalised HOSE cell line and uncultured OSE cells using β-actin as an internal control (Figure 1 and Table 1). ICAM-1 was strongly expressed in HOSE 17.1 cells, low-passage OSE cultures (data not shown) and 3 ovarian adenocarcinoma cell lines (CAOV3, 27/87 and PEO14). Moderate levels of expression were observed in the peeled OSE cells and the OVCAR3 cell line while the JAM, HEY and COLO316 cell lines expressed low levels of ICAM-1. The remaining cell lines, PEO1, OAW42 and SKOV3 did not express ICAM-1.

The expression of ICAM-1 mRNA in 2 HOSE cell lines and 9 ovarian adenocarcinoma cell lines was also examined by Northern blot analysis (Figure 2 and Table 1). There were strong and comparable levels of ICAM-1 expression in both of the HOSE cell lines. In contrast, only one of the 9 ovarian adenocarcinoma cell lines (CAOV3) expressed ICAM-1 at a similar level while another 4 cell lines (JAM, COLO316, OVCAR3 and 27/87) had much weaker levels of expression. No ICAM-1 expression was detected in the PEO1, OAW42, HEY and SKOV3 cell lines. These results were not inconsistent with those obtained by RT-PCR, although the cell lines OVCAR3 and 27/87 appeared to express more RNA by the more sensitive RT-PCR analysis than they did by Northern blot.

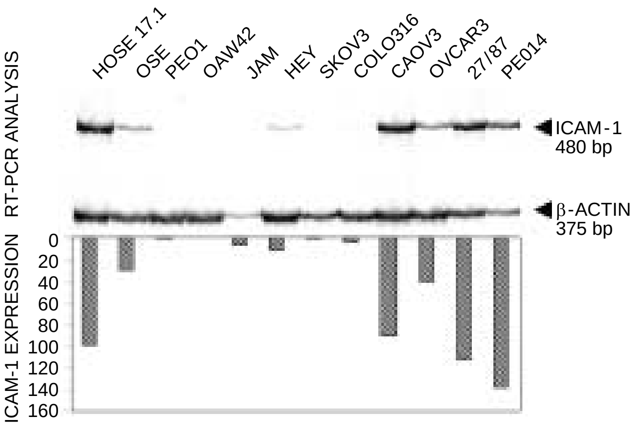

**Figure 1** Analysis of ICAM-1 expression in immortalised HOSE cells, peeled (uncultured) OSE cells and ovarian adenocarcinoma cell lines by RT-PCR. RT-PCR was carried out with β-actin as an internal control. The autoradiograph shown in the upper panel represents 29 cycles of amplification. In the lower panel, ICAM-1 expression is analysed as a percentage of β-actin levels for each lane with HOSE 17.1 set at 100%

**Table 1** ICAM-1 expression in HOSE and ovarian adenocarcinoma cell lines

| Cell line | RT-PCR percent | Northern | Immunostaining Intensity | Immunostaining Percent |
|---|---|---|---|---|
| HOSE 17.1 | 100 | XXXX | XXX | 100 |
| HOSE 1.1 | | XXXX | | |
| OSE | 30.9 | | | |
| PEO1 | 0.9 | 0 | X | 50 |
| OAW42 | 0.3 | 0 | 0 | 0 |
| JAM | 7 | X | X | 50 |
| HEY | 11.8 | 0 | 0 | 0 |
| SKOV3 | 0.8 | 0 | 0 | 0 |
| COLO316 | 5.4 | X | | |
| CAOV3 | 91.4 | XXXX | X | 10 |
| OVCAR3 | 41.4 | X | 0 | 0 |
| 27/87 | 113.1 | X | | |
| PEO14 | 138.2 | | XX | 100 |

0 = no expression detected; X, XX and XXX = increasing levels of expression.

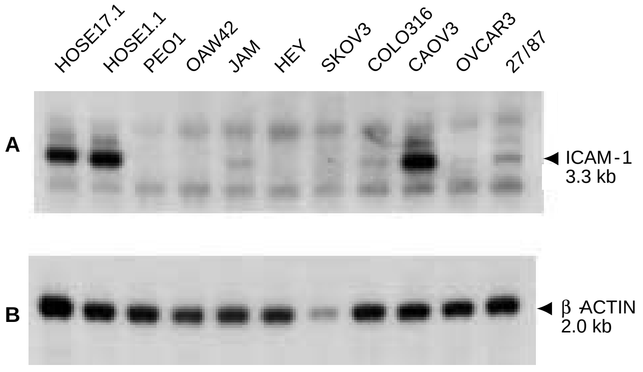

**Figure 2** Analysis of ICAM-1 expression in HOSE and ovarian adenocarcinoma cell lines by Northern blotting. Each lane represents 2 μg of polyA + RNA. (**A**) Hybridisation with the ICAM-1 probe, (**B**) hybridisation with the β-actin probe

*© 2001 Cancer Research Campaign*

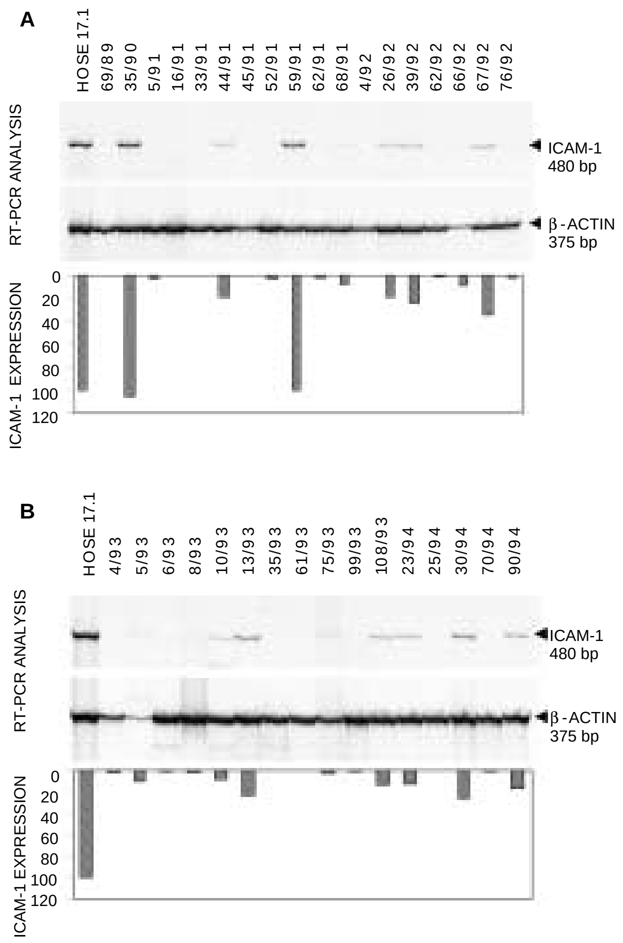

**Figure 3** Analysis of ICAM-1 expression in primary ovarian adenocarcinomas by RT-PCR. RT-PCR was carried out with β-actin as an internal control. HOSE 17.1 cells were included as a positive control. (**A**) 18 primary tumours from patients who underwent surgery from 1989 to 1992. (**B**) 16 primary tumours from patients who underwent surgery from 1993 to 1994. In each case, the autoradiograph shown in the upper panel represents 29 cycles of amplification. In the lower panel, ICAM-1 expression is analysed as a percentage of β-actin levels for each lane with HOSE 17.1 set at 100%

The levels of ICAM-1 expression were then investigated in a series of 34 primary ovarian adenocarcinomas using semi-quantitative RT-PCR (Figure 3 and Table 2). Most of the primary tumours expressed very little or no ICAM-1 RNA. There were 19 tumours (56%) which expressed less than 5% and 30 (88%) which expressed less than 25% of the amount expressed by the HOSE 17.1 cells. Only 2 tumours expressed comparable levels of ICAM-1 message to the HOSE 17.1 cells.

### Immunohistochemical analysis of ICAM-1 in ovarian cancer cell lines, normal ovaries and primary tumours

ICAM-1 expression was also examined in cell lines and primary tumours by immunohistochemistry. The HOSE 17.1 cells and a panel of 8 ovarian adenocarcinoma cell lines, for which the mRNA expression levels were known, were examined first (Table 1). Strong expression was observed in all HOSE 17.1 and PEO14 cells. There was a reduction in both the number of positive cells and the intensity of staining for the PEO1 (50% of cells positive), JAM (50% cells positive) and CAOV3 (10% cells positive) cell lines, while no staining was found in the OAW42, HEY, SKOV3 or OVCAR3 cells. When present, staining was most intense on the plasma membrane, but was also evident in the cytoplasm. This result generally correlated well with the mRNA expression data, although the CAOV3 and OVCAR3 cells expressed less protein and the PEO1 cells more protein than predicted from the RT-PCR and Northern blot data.

ICAM-1 protein expression was then investigated in 5 normal ovaries and 20 primary ovarian tumours by immunostaining (Figure 4 and Table 2). Strong expression was observed in all ovarian surface epithelial cells, including surface invaginations and epithelial inclusions present in some of the ovaries. The staining was predominantly on the plasma membrane, where it was more intense on the apical side, but was also present in the cytoplasm (Figure 4A, B). Staining of vascular endothelium was also observed.

The ICAM-1 reactivity of the primary ovarian tumours varied with respect to both percentage of positive cells and the intensity of staining (Figure 4C, D). The majority of tumours (18/20, 90%) showed a reduced intensity of staining relative to the surface epithelium of normal ovaries. There were no tumours which were completely negative, and the intensity varied from very weak to strong. The percentage of positive cells varied from 1% in the LMP tumour to 100% of epithelial cells staining. Almost 70% of cases (14/20) had fewer than half of cells staining. Many of the tumours showed a heterogeneous pattern of ICAM-1 expression with discrete groups of positive and negative cells. The majority of the tumours (16/20, 80%) showed stronger staining in the cytoplasm than on the plasma membrane (Figure 4C, D) and there was a distinct pattern of staining bordering groups of tumour cells for 6 (30%) of the tumours (Figure 4E).

Thirteen tumours were analysed by both RT-PCR and immunostaining, and there was generally a good correlation between mRNA and protein expression data for these tumours. However, there were 2 cases (6/93 and 25/94) in which little or no mRNA was detected by RT-PCR and yet were positive by immunostaining. This may have been due to the fact that different portions of the tumours were used for the RNA extraction and the immunostaining, and these may have varied in the proportion of tumour cells expressing ICAM-1.

### 5-aza-2′-deoxycytidine treatment of ovarian cancer cell lines increases ICAM-1 expression levels

To investigate whether methylation of promoter CpG islands may be involved in the down-regulation of ICAM-1 expression in ovarian adenocarcinomas, a panel of 8 ovarian adenocarcinoma cell lines were treated with 2 different concentrations of the de novo methyltransferase inhibitor 5-aza-2′-deoxycytidine. RNA was then extracted 5 days later for analysis of expression by semi-quantitative RT-PCR (Figure 5). Treatment with 0.5 μM 5-aza-2′-deoxycytidine resulted in increased ICAM-1 expression levels relative to untreated controls for all cell lines, with the greatest levels of induction occurring in the OAW42 (14.8 times), OVCAR3 (11.6 times) and JAM (6.6 times) cell lines. Treatment with 2 μM 5-aza-2′-deoxycytidine, which was toxic to the HEY cells, also resulted in increased ICAM-1 expression levels relative to untreated controls for all cell lines, but generally not to the same degree as treatment with 0.5 μM 5-aza-2′-deoxycytidine.

 © 2001 Cancer Research Campaign

**Table 2** ICAM-1 expression, 19p13 LOH status and clinico-pathological data for 45 primary ovarian adenocarcinomas

| Case | Histo | Stage | Grade | RT-PCR percent | Immunostaining | | LOH 19p13 |
|---|---|---|---|---|---|---|---|
| | | | | | Intensity | Percent | |
| HOSE 17.1 | | | | 100 | XXX | 100 | |
| OSE | | | | 30.9 | XXX | 100 | |
| 69/89 | SER | 3 | 2 | 0.3 | | | NL |
| 35/90 | SER | 3B | 2–3 | 105.2 | | | NL |
| 5/91 | SER | 3 | 2 | 3.4 | | | NL |
| 16/91 | SER | 2 | 3 | 0 | | | NL |
| 33/91 | ENDO | 4 | 3 | 0.7 | | | L |
| 44/91 | SER | 3C | 3 | 19.2 | | | NL |
| 45/91 | SER | 3C | NS | 0.4 | | | NL |
| 52/91 | SER | 2B | 2 | 3.3 | | | |
| 59/91 | SER | 3 | 3–4 | 99.6 | | | NL |
| 62/91 | SER | 3 | 3 | 2.3 | | | |
| 68/91 | SER | 3C | 3 | 8.1 | | | |
| 3/92 | SER | 3 | 3 | | XX | 0.5 | NL |
| 4/92 | SER | 3B | 3 | 1.2 | XX | 0.1 | NL |
| 26/92 | CCC | 4 | 2 | 19.4 | XX | <10% | NL |
| 39/92 | SER | 3 | 2 | 24.6 | | | NL |
| 62/92 | SER | 3C | 2–3 | 1.4 | | | L |
| 66/92 | ENDO | 3C | 3 | 9.5 | XX | 0.2 | NL |
| 67/92 | ENDO | 1C | 2–3 | 35 | | | NL |
| 76/92 | SER | 3 | 2–3 | 2.8 | | | L |
| 3/93 | SER | 3C | 3 | | | | L |
| 4/93 | SER | 3 | 1–2 | 2.2 | XX | <10% | L |
| 5/93 | SER | 3C | 2–3 | 10 | | | NL |
| 6/93 | SER | 3C | 3 | 1.3 | XX | 0.6 | L |
| 8/93 | SER | 3C | 2–3 | 2 | XXX | 0.2 | NL |
| 10/93 | SER | 3C | 2–3 | 7.8 | XXX | 0.25 | NL |
| 12/93 | CCC | 3 | 2–3 | | XX | 1 | NL |
| 13/93 | SER | 3C | NS | 23.8 | | | NL |
| 21/93 | SER | 3C | 2–3 | | XX | 1 | |
| 35/93 | SER | 3C | 3 | 0.3 | | | NL |
| 61/93 | ENDO | 4 | 2 | 0 | | | L |
| 65/93 | ENDO | 3 | 2–3 | | | | NL |
| 75/93 | SER | 2C | 3 | 2.4 | XX | 0.45 | |
| 83/93 | SER | 3 | 3 | | XX | 0.6 | |
| 99/93 | ENDO | 4 | 3 | 1.1 | X | 0.2 | NI |
| 101/93 | SER | 3C | 2 | | XX | 0.1 | |
| 108/93 | SER | 3C | 2 | 13.7 | XX | 0.2 | |
| 130/93 | SER | 3 | 2 | | XX | 0.8 | |
| 17/94 | SER | 2B | 3 | | XX | 0.3 | |
| 23/94 | SER | 3 | 3 | 12.6 | XX | 0.2 | |
| 25/94 | SER | 3C | 2 | 0 | XX | 0.4 | |
| 30/94 | MUC | OLMP1 | | 26.1 | XX | 0.01 | |
| 34/94 | SER | 3C | 3 | | | | |
| 70/94 | SER | 3C | 1–2 | 0.6 | | | L |
| 90/94 | SER | 4 | 2 | 16.9 | | | |

0 = no expression detected; X, XX and XXX = increasing levels of expression; NL = no loss; NI = not informative; L = loss; NS = not specified.

### Loss of heterozygosity analysis of the ICAM-1 locus at 19p13

ICAM-1 is located on chromosome 19 at band p13.1 to 13.2 (Ropers and Mohrenweiser, 1993). Loss of heterozygosity at 19p13 was analysed in 29 of the cases for which ICAM-1 expression had been characterised with the D19S558 microsatellite marker. LOH was detected in 8 cases (28% of informative cases). ICAM-1 expression was not associated with LOH at 19p13 for mRNA expression defined by > 1% of that of the HOSE 17.1 cells ($P = 0.32$, Fisher's exact test), but there was a weak association when mRNA expression was defined by > 10% of that of the HOSE 17.1 cells ($P = 0.05$). It was noteworthy that all tumours with LOH expressed less than 3% the level of ICAM-1 mRNA of the HOSE 17.1 cells.

### Correlation of ICAM-1 expression with histopathological characteristics and survival

There was no trend towards loss of expression with higher stage ($P = 0.5$ and 1.0 for mRNA expression defined as > 1% or > 10% that of the HOSE 17.1 cells, Test for Trend) or grade ($P = 0.59$ and 0.69, respectively, Test for Trend), nor was there any association between expression and histological subtypes ($P = 0.71$ and 0.20 respectively, $\chi^2$ test).

There was a significant correlation between ICAM-1 expression in tumour cells and survival of those patients when 10% or less of tumour cells staining was scored as negative ($P = 0.03$). However, there was no relationship between ICAM-1 expression and survival when this analysis was performed scoring 25% or less of

*© 2001 Cancer Research Campaign*

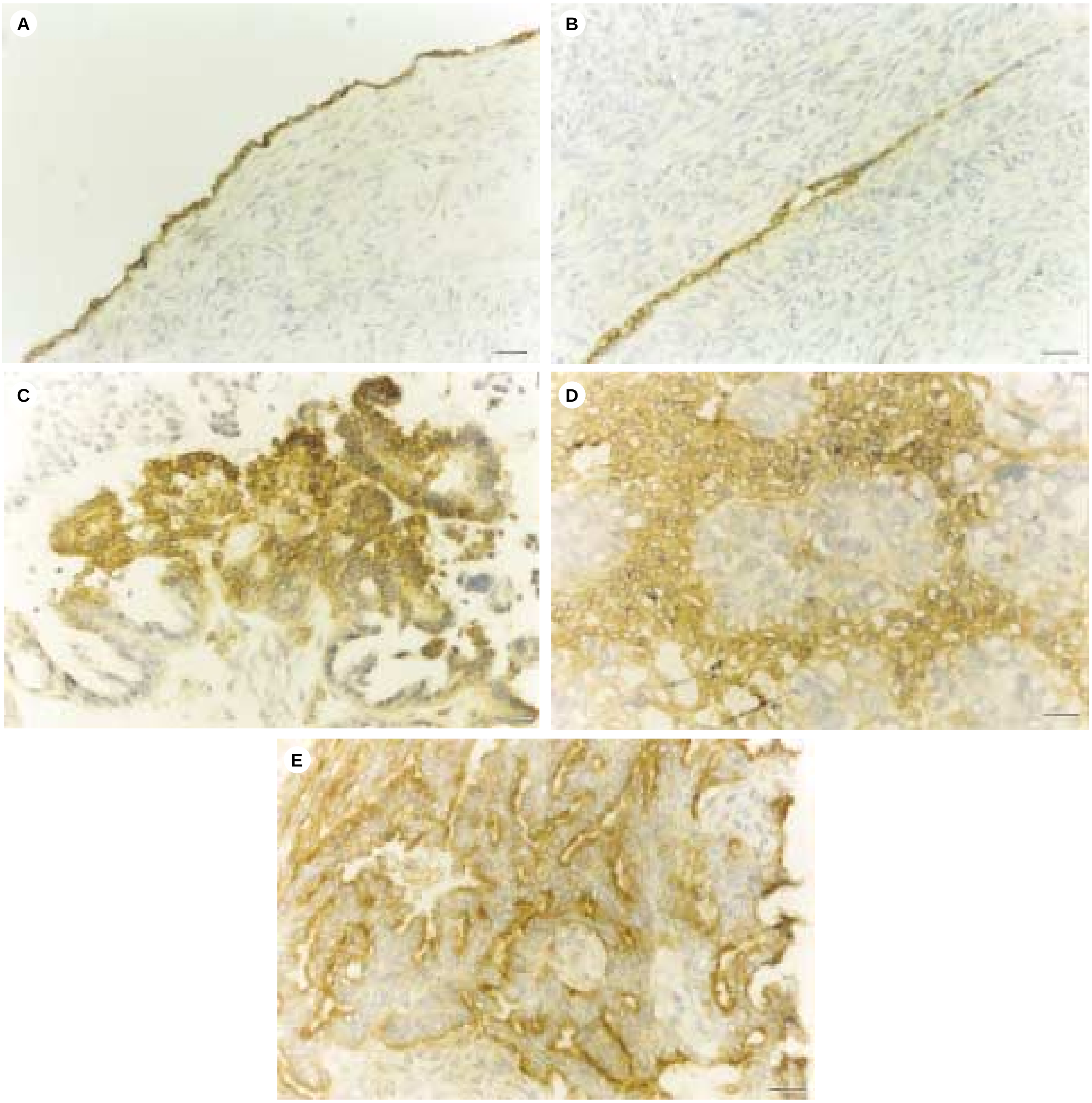

**Figure 4** Immunostaining for ICAM-1 in normal ovaries and primary ovarian tumours. (**A, B**) Normal ovaries stained for ICAM-1. Normal ovary showing strong staining (**A**) in the surface epithelium and (**B**) in a surface invagination. (**C–E**) Ovarian tumours stained for ICAM-1. (**C**) The majority of tumour cells are positive (**D**) Discrete groups of positive tumour cells. (**E**) A distinct pattern of staining bordering groups of tumour cells. Scale bar = 20 μm tumour cells staining as negative ($P$ = 0.98), nor when scoring tumours with either 1% or less ($P$ = 0.47), or 10% or less ($P$ = 0.22) of HOSE 17.1 mRNA expression as negative.

### DISCUSSION

We have analysed the expression of ICAM-1 in ovarian surface epithelial cells and cell lines, adenocarcinoma cell lines and primary tumours. ICAM-1 was expressed in normal ovarian epithelial cells, from which ovarian adenocarcinomas arise. This is in contrast to normal breast, kidney (renal tubules), gastric, pancreatic (acinar and ductal cells) and colorectal epithelia, which do not express ICAM-1 (Tomita et al, 1990; Kaiserlian et al, 1991; Koyama et al, 1992; Schwaeble et al, 1993; Shibata et al, 1996; Ogawa et al, 1998). The level of ICAM-1 expression in the immortalised HOSE cell lines was about 3-fold greater than that in the peeled OSE cells. Expression was reduced in the majority of ovarian adenocarcinoma cell lines and primary tumours at both the RNA and protein levels relative to both OSE cells and HOSE cell lines. This suggests that down-regulation of ICAM-1 expression is a common event in the development of ovarian adenocarcinoma and may be of selective advantage to these cells.

 *© 2001 Cancer Research Campaign*

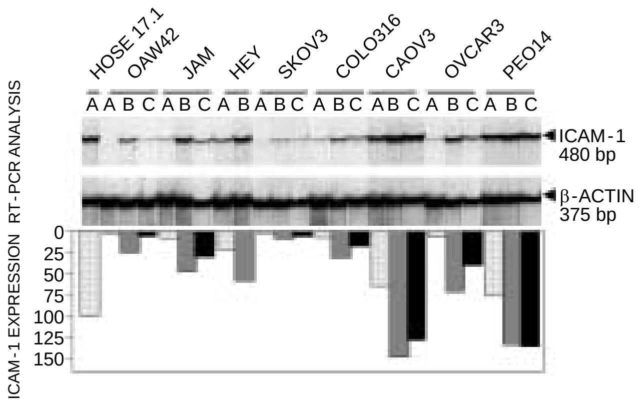

**Figure 5** Analysis of ICAM-1 expression in ovarian adenocarcinoma cell lines treated with 5-aza-2′-deoxycytidine by RT-PCR. Cell lines were treated with (**A**) 0, (**B**) 0.5 or (**C**) 2.0 μM 5-aza-2′-deoxycytidine on day 0 and harvested on day 5, followed by extraction of RNA. RT-PCR was carried out with β-actin as an internal control. The autoradiograph shown in the upper panel represents 28 cycles of amplification. In the lower panel, ICAM-1 expression is analysed as a percentage of β-actin levels for each lane with HOSE 17.1 set at 100%

Treatment of 8 cell lines with the de novo methyltransferase inhibitor 5-aza-2′-deoxycytidine dramatically increased ICAM-1 expression levels for all lines, suggesting that CpG island methylation may be a common mechanism of silencing of the ICAM-1 gene in ovarian adenocarcinoma. Extensive changes in global DNA methylation have been found in ovarian cancers (Cheng et al, 1997), and there is strong evidence that DNA hypermethylation plays a role in the inactivation of expression of E-cadherin, p16, BRCA1, GPC3 and hMLH1 in ovarian adenocarcinoma (Lin et al, 1999; McCluskey et al, 1999; Strathdee et al, 1999; Fearon, 2000). There was also frequent LOH at 19p13 (28%) at a similar rate to that reported in previous studies (Amfo et al, 1995; Wang et al, 1999). We found a weak association between this LOH and loss of ICAM-1 expression, suggesting that loss of expression may also be due to LOH in some cases.

Expression of ICAM-1 has previously been examined by immunohistochemistry in a variety of human neoplasms including breast cancer, lung cancer, squamous-cell carcinoma, renal cell cancer, pancreatic cancer, gastric cancer, ovarian adenocarcinoma, prostatic cancer, thyroid cancer and malignant melanoma (Johnson et al, 1989; Vogetseder et al, 1989; Natali et al, 1990; Tomita et al, 1990; Koyama et al, 1992; Schwaeble et al, 1993; Ogawa et al, 1998). Previous studies which have included ovarian adenocarcinomas scored either 5–30% of cells positive from a single tumour examined (Vogetseder et al, 1989) or found 0/15 primary tumours and 0/9 metastasis positive for ICAM-1 expression (Natali et al, 1990). While the antibody used in these studies was different to that used in this study, the data support our findings that the majority of ovarian tumours express little or no ICAM-1.

Some studies have found a positive correlation between ICAM-1 expression and metastatic disease in malignant melanoma (Johnson et al, 1989; Natali et al, 1990) and gastric cancer (Koyama et al, 1992). It is postulated that ICAM-1 expression on tumour cells may mediate their attachment to activated leukocytes, promote the migration of individual cells away from the primary tumour and enhance endothelial binding, extravasation and invasion into surrounding tissues (Johnson, 1991). In contrast, other investigators have reported a negative correlation between ICAM-1 expression and tumour growth and metastasis in colorectal cancer and breast cancer and found that patients with ICAM-1-positive tumours had a better prognosis than those whose tumours did not (Shibata et al, 1996; Ogawa et al, 1998). These findings may be a reflection of the different tumour backgrounds. Using the same scoring system for ICAM-1 expression as other investigators (Ogawa et al, 1998), we have also found that patients whose tumours expressed ICAM-1 had improved survival. However, as this observation is based on only 20 primary ovarian adenocarcinomas, it needs to be verified by larger independent studies.

The presence of ICAM-1 on the tumour cells may make them more susceptible to cellular immunity and indeed a significant correlation has been observed between ICAM-1 expression on tumour cells and the degree of mononuclear infiltration (Tomita et al, 1990). A number of studies have been conducted to determine the function of ICAM-1 on tumour cells in vivo. Transfection of the human *ICAM-1* gene into various cancer cells lines using plasmid or viral vectors resulted in decreased tumorigenicity when injected into mouse or rat hosts (Burno et al, 1995; Wei et al, 1996; Uzendoski et al, 1997; D'Angelica et al, 1999; Kikuchi et al, 1999). Several lines of evidence demonstrate that this protection was due, at least in part, to activation of cellular immunity by ICAM-1 (Burno et al, 1996; Wei et al, 1996; Uzendoski et al, 1997; D'Angelica et al, 1999). Therefore, while more investigation is required to determine the significance of the reduced ICAM-1 expression levels in ovarian cancer, evidence from other models suggests that *ICAM-1* gene transfer may be of value for the immunotherapy of ovarian cancer.

### ACKNOWLEDGEMENTS

We gratefully acknowledge the support of the National Health and Medical Research Council of Australia and the Queensland Cancer Fund. We thank Dr Samuel Mok for the kind donation of the HOSE cell lines, Terry Hurst for culturing the OSE cells, and Michael Walsh for assistance with the immunohistochemistry.

### REFERENCES

Amfo K, Neyns B, Teugels E, Lissens W, Bourgain C, De Sutter P, VanDamme B, Vamos E and De Greve J (1995) Frequent deletion of chromosome 19 and a rare rearrangement of 19p13.3 involving the insulin receptor gene in human ovarian cancer. *Oncogene* **11**: 351–358

Arnold JM, Mok SC, Purdie D and Chenevix-Trench G (2001) Decreased expression of the Id3 gene at 1p36.1 in ovarian adenocarcinomas. *Br J Cancer* **84**: 1–8

Buick RN, Pullano R and Trent JM (1985) Comparative properties of five human ovarian adenocarcinoma cell lines. *Cancer Res* **45**: 3668–3676

Burno DK, Kyprianou N, Sartor W, Fabian DF, Turner J, Vu T, Patel A, Trimbach C and Lefor AT (1995) Transfection of a murine fibrosarcoma with intercellular adhesion molecule-1 enhances the response to adoptive immunoptherapy. *Surgery* **118**: 237–244

Burno DK, Fabian DF and Lefor AT (1996) ICAM-1 increases in vitro adhesion and cytotoxicity in a murine fibrosarcoma. *J Surg Res* **60**: 398–402

Chenevix-Trench G, Leary J, Kerr J, Michel J, Kefford R, Hurst T, Parsons P, Friedlander M and Khoo SK (1992) Frequent loss of heterozygosity on chromosome 18 in ovarian adenocarcinoma which does not always include the DCC locus. *Oncogene* **7**: 1059–1065

Cheng P, Schmutte C, Cofer KF, Felix JC, Yu MC and Dubeau L (1997) Alterations in DNA methylation are early, but not initial, events in ovarian tumorigenesis. *Br J Cancer* **75**: 396–402

D'Angelica M, Tung C, Allen P, Halterman M, Delman K, Delohery T, Klimstra D, Brownlee M, Federoff H and Fong Y (1999) Herpes simplex virus (HSV)-mediated ICAM-1 gene transfer abrogates tumorigenicity and induced anti-tumour immunity. *Mol Med* **5**: 606–616

Diamond MS, Staunton DE, Marlin SD and Springer TA (1991) Binding of the integrin Mac-1 (CD11b/CD18) to the third immunoglobulin-like domain of ICAM-1 (CD54) and its regulation by glycosylation. *Cell* **65**: 961–971

© 2001 Cancer Research Campaign 

Fearon ER (2000) BRCA1 and E-cadherin promoter hypermethylation and gene inactivation in cancer-association or mechanism? *J Natl Cancer Inst* **92**: 515–517

Fogh J and Trempe G (1975) New human cell lines. *In*: Fogh J (ed) *Human tumour cells in vitro*, pp 155–159, Plenum: New York

Hamilton TC, Young RC, McKoy WM, Grotzinger KR, Green JA, Chu EW, Whang-Peng J, Rogan AM, Green WR and Ozols RF (1983) Characterization of a human ovarian carcinoma cell line (NIH:OVCAR-3) with androgen and estrogen receptors. *Cancer Res* **43**: 5379–5389

Johnson JP (1991) Cell adhesion molecules of the immunoglobulin supergene family and their role in malignant transformation and progression to metastatic disease. *Cancer Metastasis Rev* **10**: 11–22

Johnson JP, Stade BG, Holzmann B, Schwable W and Riethmuller G (1989) De novo expression of intercellular-adhesion molecule 1 in melanoma correlates with increased risk of metastasis. *Proc Natl Acad Sci USA* **86**: 641–644

Kaiserlian D, Rigal D, Abello J and Revillard JP (1991) Expression, function and regulation of the intercellular adhesion molecule-1 (ICAM-1) on human intestinal epithelial cell lines. *Eur J Immunol* **21**: 2415–2421

Kikuchi T, Joki T, Akasaki Y, Abe T and Ohno T (1999) Induction of antitumour immunity using intercellular adhesion molecule-1 (ICAM-1) transfection in mouse glioma cells. *Cancer Lett* **142**: 201–206

Koyama S, Ebihara T and Fukao K (1992) Expression of intercellular adhesion molecule 1 (ICAM-1) during the development of invasion and/or metastasis of gastric carcinoma. *J Cancer Res Clin Oncol* **118**: 609–614

Kruk PA, Maines-Bandiera SL and Auersperg NA (1990) Simplified method to culture ovarian surface epithelium. *Lab Invest* **63**: 132–136

Langdon SP, Lawrie SS, Hay FG, Hawkes MM, McDonald A, Hayward IP, Schol DJ, Hilgers J, Leonard RCF and Smyth JF (1988) Characterization and properties of nine human ovarian adenocarcinoma cell lines. *Cancer Res* **48**: 6166–6172

Lin H, Huber R, Schlessinger D and Morin PJ (1999) Frequent silencing of the GPC3 gene in ovarian cancer cell lines. *Cancer Res* **59**: 807–810

McCluskey LL, Chen C, Delgadillo E, Felix JC, Muderspach LI and Dubeau L (1999) Differences in p16 gene methylation and expression in benign and malignant ovarian tumours. *Gynecol Oncol* **72**: 87–92

Natali P, Nicotra MR, Cavaliere R, Bigotti A, Romano G, Temponi M and Ferrone S (1990) Differential expression of intercellular adhesion molecule 1 in primary and metastatic melanoma lesions. *Cancer Res* **50**: 1271–1278

Ogawa Y, Hirakawa K, Nakata B, Fujihara T, Sawada T, Kato Y, Yoshikawa K and Sowa M (1998) Expression of intercellular adhesion molecule-1 in invasive breast cancer reflects low growth potential, negative lymph node involvement, and good prognosis. *Clin Cancer Res* **4**: 31–36

Parkin DM, Pisani P and Ferlay J (1999) Estimates of the worldwide incidence of 25 major cancers in 1990. *Int J Cancer* **80**: 827–841

Ropers HH and Mohrenweiser H (1993) Report of the committee on the genetic constitution of chromosome 19. *Genet Prior Rep* **1**: 524–547

Sambrook J, Fritch EF and Maniatis T (1989) *Molecular cloning: A laboratory manual, second edition*. Cold Spring Harbour Laboratory Press; New York

Schwaeble W, Kerlin M, Meyer Zum Buschenfelde K and Dippold W (1993) De novo expression of intercellular adhesion molecule-1 (ICAM-1, CD54) in pancreas cancer. *Int J Cancer* **53**: 328–333

Shibata M, Ando K, Amano S and Kurosu Y (1996) Local expression and circulating form of ICAM-1 in colorectal cancer. *Ann. Cancer Res. Ther* **5**: 29–33

Springer TA Adhesion receptors of the immune system (1990) Nature **346**: 425–434

Springer TA, Dustin ML, Kishimoto TK and Marlin SD (1987) The lymphocyte-function-associated LFA-1, CD2 and LFA-3 molecules: Cell-adhesion receptors of the immune system. *Ann Rev Immunol* **5**: 223–252

Strathdee G, MacKean MJ, Illand M and Brown R (1999) A role for methylation of the hMLH1 promoter in loss of hMLH1 expression and drug resistance in ovarian cancer. *Oncogene* **18**: 2335–2341

Tomita Y, Nishiyama T, Watanabe H, Fujiwara M and Sato S (1990) Expression of the intercellular adhesion molecule-1 (ICAM-1) on renal-cell cancer: possible significance in host immune responses. *Int J Cancer* **46**: 1001–1006

Tsao S-W, Mok SC, Fey EG, Fletcher JA, Wan TSK, Chew E-C, Muto MG, Knapp RC and Berkowitz RS (1995) Characterization of human ovarian surface epithelial cells immortalized by human papilloma viral oncogenes (HPV-E6E7 ORFs). *Exp Cell Res* **218**: 499–507

Uzendoski K, Kantor JA, Abrams SI, Schlom J and Hodge JW (1997) Construction and characterization of a recombinant vaccinia virus expressing murine intercellular adhesion molecule-1: induction and potentiation of antitumour responses. *Hum Gene Ther* **8**: 851–860

Vogetseder W, Feichtinger H, Schulz TF, Schwaeble W, Tabaczewski P, Mitterer M, Bock G, Marth C, Dapunt O, Mikuz G and Dierich MP (1989) Expression of 7F7-antigen, a human adhesion molecule identical to intercellular adhesion molecule-1 (ICAM-1) in human carcinomas and their stromal fibroblasts. *Int J Cancer* **43**: 768–773

Wang Z-J, Churchman M, Campbell IG, Xu W-H, Yan Z-Y, McCluggage WG, Foulkes WD and Tomlinson IPM (1999) Allele loss and mutation screen at the Peuta-Jeghers (LKB1) locus (19p13.3) in sporadic ovarian tumours. *Br J Cancer* **80**: 70–72

Ward BG, Wallace K, Shepherd JH and Balkwill FR (1987) Intraperitoneal xenografts of human epithelial ovarian cancer in nude mice. *Cancer Res* **47**: 2662–2667

Wei K, Wilson JG, Jurgensen CH, Iannone MA, Wolberg G and Huber BE (1996) Xenogeneic ICAM-1 gene transfer suppresses tumourigenicity and generates protective antitumour immunity. *Gene Ther* **3**: 531–541

Wilson AP (1984) Characterization of a cell line derived from the ascites of a patient with papillary serous cystadenocarcinoma of the ovary. *J Natl Cancer Inst* **72**: 513–520

Wolf CR, Hayward IP, Lawrie SS, Buckton K, McIntyre MA, Adams DJ, Lewis AD, Scott ARR and Smyth JF (1987) Cellular heterogeneity and drug resistance in two ovarian adenocarcinoma cell lines derived from a single patient. *Int J Cancer* **39**: 695–702

Woods LK, Morgan RT, Quinn LA, Moore GE, Semple TU and Stedman KE (1979) Comparison of four new cell lines from patients with adenocarcinoma of the ovary. *Cancer Res* **39**: 4449–4459

Wong AST, Maines-Bandiear SL, Rosen B, Wheelock MJ, Johnson KR, Leung PCK, Roskelley CD and Auersperg N (1999) Constitutive and conditional cadherin expression in cultured human ovarian surface epithelium: influence of family history on ovarian cancer. *Int J Cancer* **81**: 180–188

*© 2001 Cancer Research Campaign*
